# A humanized anti-DLL4 antibody promotes dysfunctional angiogenesis and inhibits breast tumor growth

**DOI:** 10.1038/srep27985

**Published:** 2016-06-15

**Authors:** Xuelian Jia, Wenyi Wang, Zhuobin Xu, Shijing Wang, Tong Wang, Min Wang, Min Wu

**Affiliations:** 1 State Key Laboratory of Natural Medicines, School of Life Science & Technology, China Pharmaceutical University, Nanjing, Jiangsu 210009, China; 2 The Rutgers Center for Computational and Integrative Biology, Rutgers University, Camden, NJ 08102, United States

## Abstract

Blockage of Delta-like 4 (DLL4)-directed Notch signaling induces excessive tip cell formation and endothelial proliferation resulting in dysfunctional angiogenesis in tumors. MMGZ01, as a murine anti-human DLL4 monoclonal antibody, specifically binds to human DLL4 and blocks Notch pathway. Here, the structure of MMGZ01 variable fragment (Fv) was established and framework region (FR) residues which supported complementarily determining region (CDR) loop conformation were identified. Important residues interactions were also identified through docking MMGZ01 Fv with antigen epitope in DLL4. To humanize the murine antibody, we modified MMGZ01 Fv through CDR grafting and the reconstructed antibody (H_3_L_2_) maintained similar structure and binding affinity to parental MMGZ01 after back mutation of 12 canonical murine residues in the FRs. Meanwhile, H_3_L_2_ promoted human umbilical vein endothelial cell (HUVEC) proliferation through inhibiting DLL4-directed Notch pathway. Moreover, in MDA-MB-231-bearing nude mice, H_3_L_2_ induced dysfunctional angiogenesis and tumor cell apoptosis and showed superior anti-tumor activity. In conclusion, H_3_L_2_ is an ideal humanized antibody that inhibits tumor growth through targeting DLL4-Notch pathway and has attracting potentials for clinical applications.

The Notch signaling pathway is implicated with cell fate decision, stem cell renewal and differentiation in many postnatal tissues during development^1^. In mammalian, it consists of four receptors (Notch 1–4) and five canonical ligands (Jagged 1, 2 and Delta-like 1, 3, 4). Overexpressed Notch receptors or ligands activates the Notch-mediated tumorigenesis pathway through deregulating in many hematological malignancies and solid tumors^1^,^2^.

As a key ligand of Notch pathway, delta-like 4 (DLL4) is endothelium-specific with higher expression in tumor vasculature compared to normal tissues^3^. Through regulating the proliferation of tip cells in sprouting vessels, DLL4 directs the generation of functional and organized vasculatures in tumors^4^,^5^. Further blockage of DLL4 increases immature capillary sprouting and branching due to excessive tip cell formation and endothelial proliferation, but with reduced vessel lumen size. These dysfunctional vasculatures perform poor perfusion, thus increase hypoxia in tumors^6^,^7^. Many DLL4/Notch inhibitors have been developed with ideal anti-tumor efficiency in pre-clinical models or clinical trials^2^,^8^,^9^.

In a previous study, we developed a murine anti-human DLL4 monoclonal antibody with expected anti-angiogenesis and anti-tumor effects^10^. While murine antibody has the advantage of high antigen binding affinity and specificity, the clinical usefulness of such antibody is still limited to the human anti-mouse antibody (HAMA) response^11^,^12^. Chimeric antibodies which graft variable regions of murine antibodies onto human constant regions are still likely to induce severe immune response^13^,^14^. Thus, humanization of murine antibody variable regions becomes a prospective alternative. Jones *et al*.^15^ firstly grafted complementarily determining regions (CDRs) in heavy and light chain variable regions (VH, VL) of murine antibody B1–8 onto human antibody and generated a CDR grafted antibody, which possessed comparable affinity with B1–8. However, compared to their parental mouse antibodies, many CDR grafted antibodies present lower antigen binding affinities due to the lack of framework regions (FRs) canonical residues which supported CDR loop conformation^16^.

In this study, we firstly chose suitable human V and J donors for CDR grafting based on a precisely modeled MMGZ01 variable fragment (Fv) structure. Through back mutation of 7 canonical residues in VH and 5 canonical residues in VL to the original murine one, the further designed version (H_3_L_2_) achieved comparable DLL4 binding affinity and specificity with the murine-human chimeric antibody (H_C_L_C_). Comprehensive *in vivo* and *in vitro* evaluation revealed that H_3_L_2_ was able to inhibit tumor growth and disturb tumor angiogenesis.

## Results

### Antibody modeling and evaluation

The amino acid sequences of MMGZ01 VH, VL were loaded to Molecular Operating Environment (MOE) software, version 2013. 08, and FRs or CDRs in VH and VL were identified by Kabat numbering scheme^17^. Antibody structures in Protein Data Bank (PDB)^18^ were searched and scored based on sequence similarity as well as structural fitness, in particular the backbone integrity, which was evaluated based on a structural scoring scheme developed by MOE. The first task in antibody modeling is to find FRs templates for VH and VL, where, the best scored FRs templates are normally from different antibodies. We comprehensively selected FRs templates from a single antibody 1E6O (see Table 1), in order to get a Fv dimer framework with suitable VH-VL orientation.

**Figure 1:**
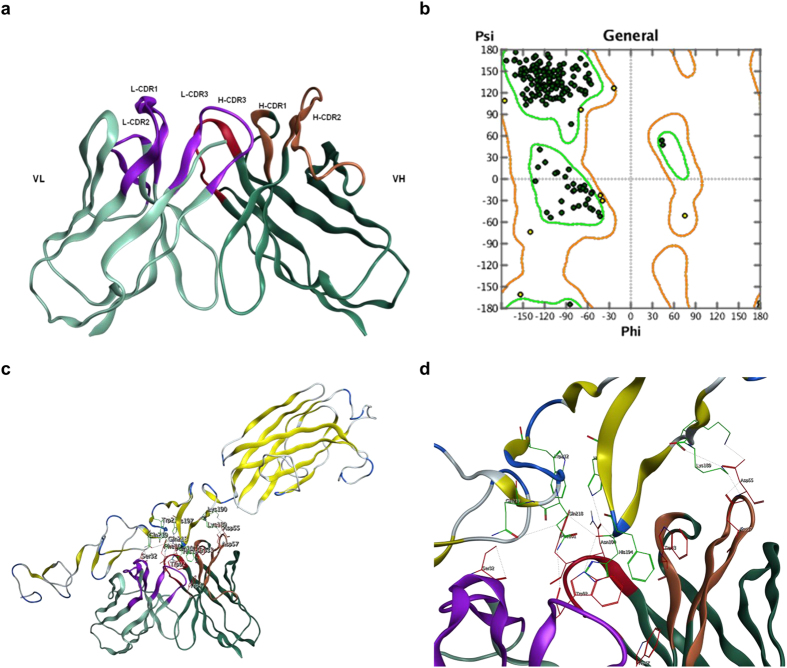
Structure model of MMGZ01 Fv and complex structure with DLL4. (**a**) 3D structure of the simulated MMGZ01 Fv. The structure was built in MOE with various antibody structures templates found in PDB database. (**b**) Structure evaluation with Ramachandran plot. Residues in the core regions are rendered green and residues in allowed regions are rendered yellow. (**c**) Complex structure of MMGZ01 Fv and DLL4 predicted by molecular docking. (**d**) Residues interaction in antibody-antigen binding site. Interactive residues located in antibody are rendered red and those located in DLL4 are rendered green. DLL4 **K189** had hydrogen bond with HCDR2-D57, ion bond with HCDR2-D55 and D57. DLL4 **K190** had ion bond with HCDR2-55D. DLL4 **H194** had hydrogen bond with LCDR3-W92 and HCDR3-N100. DLL4 **F195** had hydrophobic interaction with LCDR3-W92, HCDR1-W33 and HFR2-W47. DLL4 **H197** had hydrogen bond with HCDR3-N100. DLL4 **W212** had hydrophobic interaction with HCDR3-F101. DLL4 **K218** had hydrogen bond with LCDR3-W92. DLL4 **Q219** had hydrogen bond with LCDR1-S32.

As templates for the VH-VL framework were determined, the CDR loops templates were further assigned based on loop length and similarity prior to the grafting of CDRs onto the F_V_ dimer framework. In loop grafting, multiple templates were combined, aligned and superposed at the backbone atoms of corresponding residues in the FRs. The CDR residues of CDR templates were bonded to the FRs template and redundant residues were deleted. Four rounds of tethered energy minimization were then applied to relieve strained geometry and atom clashes in the newly composed antibody subdomain. With these steps, a precise structure of MMGZ01Fv was built automatically by MOE Antibody Builder module (Fig. 1a).

A Ramachandran plot was displayed to check the stereo-chemical quality of established Fv structure^19^. Residues were rendered in green if they located in the core region, yellow if in the allowed region and red if in the outlier region. As shown in Fig. 1b, all the residues distributed in the allowed regions, indicating that the established Fv structure is reasonable.

### Epitope mapping

To identify the epitopes recognized by MMGZ01 and to minimize the possible poses during antibody-antigen docking, a dodecapeptide phage display library was screened^20^. Phage-peptides bound to MMGZ01 were panned and enriched for 4 cycles, followed by monoclonal Enzyme-linked immunosorbent assay (ELISA). The phage clones gave ELISA signal 3-fold of control (3% Bull Serum Albumin, BSA) were DNA sequenced and aligned for consensus motif. As shown in Supplementary Fig. S1, the consensus residues between positive clones were KK—HF-H. These residues were found in DLL4 (residues 189–197) and were likely to constitute a conformational epitope.

### Complex structure of MMGZ01-DLL4 predicted by molecular docking

Structure model of DLL4 (residue range from 27 to 283) was provided by SWISSMODEL, based on template structure in PDB (PDB ID 4xlwB, sequence identity 80%). Before docking, the epitope residues mapped by phage display library screening were selected as contact residues in DLL4. A rigid receptor docking program ZDOCK^21^,^22^ was used, and the output files gave top 10 modeled prediction of complex structure. The top one complex structure was shown in Fig. 1c, and the residues interactions between antigen and antibody binding site were shown in Fig. 1d. There were hydrogen bond, ion bond or hydrophobic interactions between DLL4 residues: K189, K190, H194, F195, H197, W212, Q218, Q219 and MMGZ01 residues: HCDR1-W33, HFR2-W47, HCDR2-D55, HCDR2-D57, HCDR3-N100, HCDR3-F101, LCDR1-S32, LCDR3-W92. The interactive MMGZ01 residues were mostly located in CDRs excepted HFR2-W47, which was recommended to be retained in humanization.

### Humanization of MMGZ01 Fv

With the precise structure of MMGZ01 Fv, we further identified the canonical residues in FRs which were important for CDR conformation maintaining or binding affinity of antibody. These canonical residues in FRs were shown in Table 2 and identified as 3 types:1. VH-VL interface core residues: the major Fv dimer contact residues which may affect the orientation of VH and VL domains and indirectly affect antigen binding^23^; 2. CDR loop foundation residues: residues close to the CDR loops and buried in the VH or VL, which are important for CDR conformations maintaining; 3. CDR loop interaction residues: residues have hydrogen bond, ionic bond or hydrophobic interaction with CDR loop residues. These canonical residues were considered to be retained from the murine antibody when CDR grafting.

**Figure 2:**
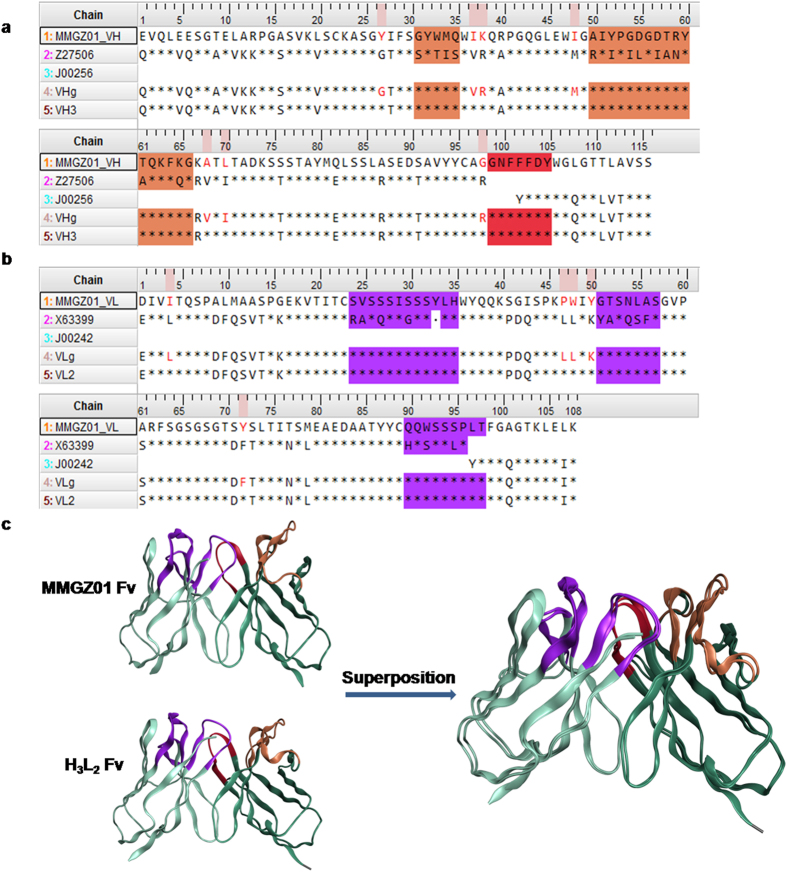
Amino acid sequences alignment and structure comparison between murine and humanized antibody. (**a**) VH sequence alignment and (**b**) VL sequence alignment. MMGZ01_VH and MMGZ01_VL were named for VH and VL regions of MMGZ01 murine antibody. Z27506 and J00256 found in V and J genes were chosen as human FRs donors for the humanized VH. Similarly, X63399 and J00242 were chosen as human FRs donors for the humanized VL. VH_g_ and VL_g_ were VH and VL regions of CDR grafted antibody. VH_3_ and VL_2_ were VH and VL regions of the final version of humanized antibody. The residues colored red were back mutations in the final version. * represents residues that are identical to the corresponding residues of MMGZ01. CDR1-2 regions of VH are marked orange and CDR3 region of VH are marked red. CDR1-3 regions of VL are marked purple. CDR regions were identified with the Kabat numbering scheme^17^. (**c**) Superposed structure of MMGZ01 Fv and H_3_L_2_ Fv. The calculated structural RMSD was 0.824 Å.

To get a CDR grafted Fv, we searched human immunoglobulin germline V and J genes for FR donors based on sequence similarity and most canonical residues reserved. As shown in Fig. 2, human germline V gene IGHV1-69*02 was selected as donor for FR1-3 and J gene IGHJ4 for FR4 of VH. Similarly, human germline V gene IGKV6-21*01 was selected for FR1-3 and J gene IGKJ2 for FR4 of VL. After grafting CDRs of MMGZ01 VH and VL onto FRs of human donors, we got a CDR grafted Fv (VH_g_, VL_g_).

Aligned with MMGZ01 VH, VL, there were still 7 canonical residues differed in VH_g_, and 5 differed in VL_g_ (Supplementary Fig. S2). Meanwhile, results of ELISA showed that the CDR grafted antibody failed to reach the target affinity (Supplementary Fig. S3), emphasizing the importance of FR residues, in particular the 12 canonical residues in maintaining the binding affinity. Back mutations of these 12 canonical residues to the CDR grafting were essential in binding affinity recovery. Considering the principle of humanization - reducing immunogenicity, we expected to mutate less residues while retaining high affinity. Thus, the importance of each of these 12 canonical residues to the MMGZ01 Fv structure was further evaluated by site-directed mutagenesis and prediction of stability changes^24^,^25^. If the mutations altered the stability too much, they may cause the loss of the antibody’s structure and function. In each mutation, the murine residue were mutate to the corresponding one in VH_g_ or VL_g_, and change in stability from mutant antibody to the wild type was reported and expressed as dStability. When generating an ensemble, dStability is equal to the Boltzman average of the relative stabilities of the ensemble. As shown in Supplementary Table S1, a more positive dStability value indicated a more unstable mutation^24^,^25^, and these canonical residues were recommended to back mutate to the murine one after CDR grafting.

From the above, canonical residues belonged to all the 3 types and/or with dStability most positive were extremely important and back mutated to the corresponding murine one with priority, residues less important were gradually added and back mutated in the latter version (see Supplementary Fig. S2). Through transient transfection, the humanized antibodies were expressed in CHO-s cell, and the binding activity was analyzed and compared by ELISA assay. As shown in Supplementary Fig. S3, the humanized antibody (H_3_L_2_) which back mutated all the 12 residues possessed the most similar antigen binding activity with the chimeric antibody H_C_L_C_. The amino acid sequences of H_3_L_2_ were shown in Fig. 2a,b.

### Structural comparison between MMGZ01 and H_3_L_2_ Fv

Superposition is a useful tool for comparing protein structures and the RMSD value reflects conformation difference^26^. The 3-D structure of H_3_L_2_ Fv was modeled as described above, structure templates and sequence similarities were shown in Supplementary Table S2. Then the structure of H_3_L_2_ Fv were superposed with the parent MMGZ01 Fv structure and structural Root Mean Square Deviation (RMSD) were calculated. The resulting superposition model was shown in Fig. 2c, and the RMSD value between the superposed Fv structures was 0.824 Å. The results indicates that H_3_L_2_ Fv had a similar conformation with the parent MMGZ01 Fv.

### Stable expression and purification of H_C_L_C_ and H_3_L_2_

**Figure 3:**
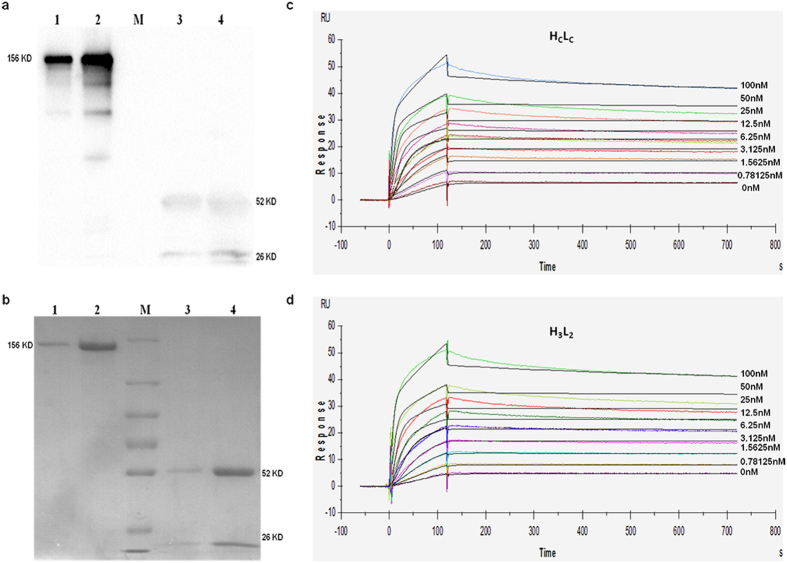
Expression, purification and antigen binding kinetics of chimeric or humanized antibody. (**a**) SDS-PAGE and (**b**) Western Blot assay of stable expressed and purified H_C_L_C_/H_3_L_2_. Lane 1 and 3 were non-reduced and reduced H_C_L_C_, lane 2 and 4 were non-reduced and reduced H_3_L_2_ respectively. Lane M were marker with molecular weight of 250, 130, 100, 70, 55, 35, 25 KDa. (**c,d**) Antigen binding kinetics analyzed by SPR. Sensorgrams of series concentrations of rhDLL4 binding to (**c**) H_C_L_C_ or (**d**) H_3_L_2_ were recorded and fitted with a 1:1 binding model. *k*_*d*_ and *k*_*a*_ of H_C_L_C_ were 2.42 × 10^−5^ 1/s and 1.72 × 10^7^ 1/Ms, and *K*_*D*_ calculated from *kd*/*ka* was 1.14 × 10^−12^ M. R^2^ value of H_c_L_c_ was 1.44 and R_max_ was 50.77 RU. *k*_*d*_ and *k*_*a*_ of H_3_L_2_ were 2.59 × 10^−5^ 1/s and 1.15 × 10^7^ 1/Ms, and *K*_*D*_ was 2.26 × 10^−12^ M. R^2^ value of H_3_L_2_ was 1.68 and R_max_ was 50.9.

After 2-round screening of stable transfectants^27^, we finally got 4 clones with highest expression levels of H_C_L_C_ or H_3_L_2_. Clones produced about 5 mg/L H_C_L_C_ or 7 mg/L H_3_L_2_ were cultured and supernatant was purified by Protein A affinity chromatography. The purified protein was analyzed by Western Blot and SDS-PAGE. As shown in Fig. 3a,b, H_C_L_C_ and H_3_L_2_ shared the expected molecular weight of 156 KDa in non-reduced condition and 52/26 KDa in reduced condition.

### Affinity of H_3_L_2_ to rhDLL4

To better evaluate the binding affinity and kinetics of H_3_L_2_ to rhDLL4, an SPR-based assay was used. H_C_L_C_ or H_3_L_2_ was captured by a capture antibody immobilized onto the biosensor chip surface. Then gradient concentrations of rhDLL4 up to 100 nM were passed through the surface. The recorded sensorgrams were fitted with a 1:1 binding model (Fig. 3c,d), and the equilibrium constant *K*_*D*_ of H_3_L_2_ calculated from ratio of *k*_*d*_*/k*_*a*_ was 2.26 × 10^−12^ M, which is comparable to that of H_C_L_C_ (1.41 × 10^−12^ M). The humanized antibody H_3_L_2_ exhibited high affinity and specificity to rhDLL4 which were similar to that of its parental antibody.

### Binding capacity to HUVEC cells

**Figure 4:**
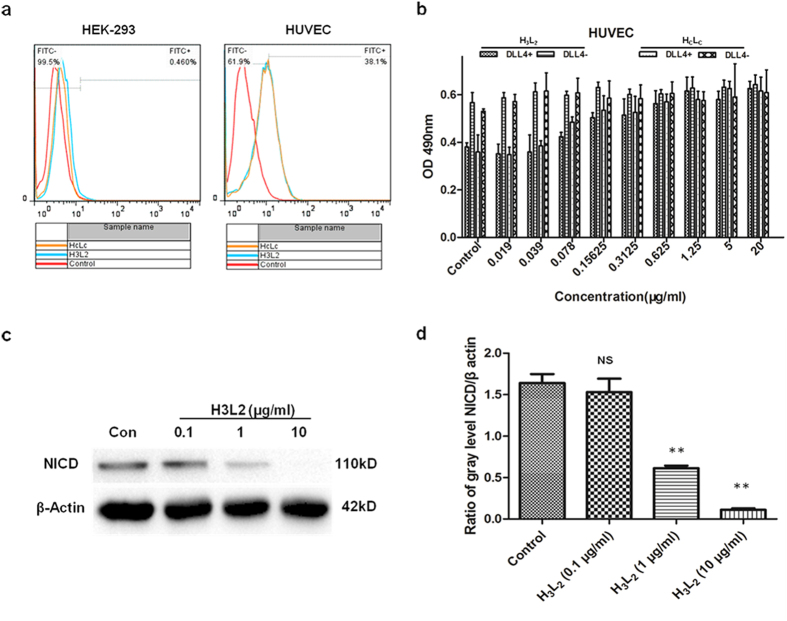
Effects of H_3_L_2_ on HUVEC cells. (**a**) Binding capacity to HUVEC cells analyzed by Flow Cytometry. H_3_L_2_ and H_C_L_C_ exhibited similar binding rates to HUVEC cells (36.9% and 38.1% respectively), and low binding to HEK-293 was observed (1.72% and 0.46% respectively). (**b**) H_3_L_2_ promoted HUVEC cells proliferation in a dose dependent manner. Cell proliferation were measured by MTT method and shown as absorbance units (mean ± SD, n = 3). (**c**,**d**) NICD levels in HUVEC cells detected by Western Blot. H_3_L_2_ inhibited Notch1 activation in a dose dependent manner. (**d**) Ratio of NICD and β actin gray levels. Gray levels were analyzed by Alpha Ease FC^TM^ software, version 3.1.2. Data are given as the mean ± SD (n = 3). NS: no significance, **p ≤ 0.01 versus Control.

HUVEC is an endothelial cell line shown to express DLL4 ligand^28^. To investigate if H_3_L_2_ could bind to DLL4 ligand natively expressed on cells, we used Flow Cytometry to detect binding capacity of H_3_L_2_ to HUVEC cells. As shown in Fig. 4a, binding rates of 10 μg/ml H_3_L_2_ or H_C_L_C_ with HUVEC cells were 36.9% and 38.1% respectively. Low binding of H_3_L_2_ or H_C_L_C_ to the DLL4 negative cell line HEK-293 was observed (1.72%, 0.46%). This results indicates that H_3_L_2_ specifically bound to DLL4 expressed on HUVEC cells, and its binding capacity was comparable to that of H_C_L_C_.

### H_3_L_2_ promotes HUVEC cells proliferation through inhibition of DLL4 directed Notch signaling

Prior to HUVEC cells seeding, rhDLL4 was immobilized onto 96-well plates to simulate Notch receptor of adjacent cells^29^,^30^. rhDLL4 caused growth inhibition of HUVEC cells compared to plates without rhDLL4, and H_3_L_2_ reversed this inhibition in a dose dependent manner (Fig. 4b). Notch intracellular domain (NICD) levels were analyzed as representative of Notch signaling activation^4^. In a Western Blot assay, H_3_L_2_ led to significant NICD reduction in HUVEC cells in a dose dependent manner, and resulted in almost NICD elimination at concentration of 10 μg/ml (*P* = 0.000114) (Fig. 4c,d). These results indicates that proliferation of HUVEC cells caused by H_3_L_2_ was due to inhibition of DLL4 directed Notch signaling.

### H_3_L_2_ modulates angiogenesis and inhibit tumor growth

**Figure 5:**
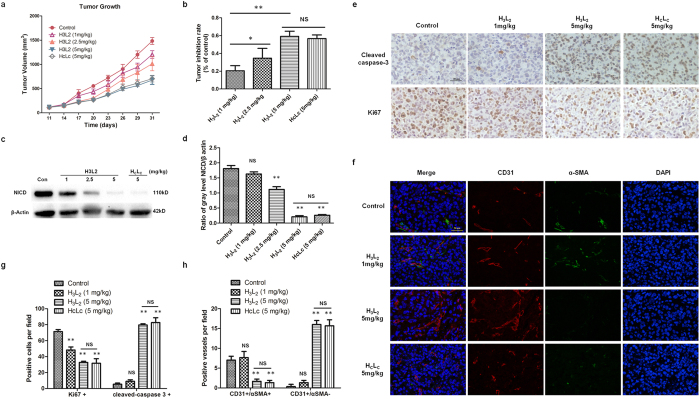
The *in vivo* anti-tumor activity of H_3_L_2_ in the MDA-MB-231 xenograft model. (**a**) Tumor growth curves of nude mice in different groups. 30 nude mice were randomly divided into 5 groups for drug administration on the 11th day post tumor cell inoculation, tumor volumes of mice were measured with the mean ± SD, n = 6. (**b**) Tumor inhibition rates of different groups. Average tumor inhibition rates of each group were 19% (H_3_L_2_ 1 mg/kg), 34% (H_3_L_2_ 2.5 mg/kg), 59% (H_3_L_2_ 5 mg/kg) and 56% (H_C_L_C_ 5 mg/kg). Data are given as the mean ± SD (n = 6). (**c**) NICD levels in tumor cells analyzed by Western Blot. (**d**) Ratio of NICD and β actin gray levels. Gray levels were analyzed by Alpha Ease FC^TM^ software, version 3.1.2. Data are given as the mean ± SD (n = 3). (**e**) Expression levels of Ki67, cleaved caspase-3 (brown staining) in tumors analyzed by IHC. Photomicrographs were taken at 400x magnifications. Scale bar = 50 μm. (**g**) Quantifications of Ki67 or cleaved caspase-3 positive cells per field. Data are given as the mean ± SD (n = 3). (**f**) Immunofluorescence double staining of CD31-positive (red fluorescence) and α-SMA-positive (green fluorescence) cells in tumors. Scale bar = 50 μm. (**h**) Quantifications of mature (CD31+/α SMA+) or immature (CD31+/α SMA−) vessels per field. Data are given as the mean ± SD (n = 3). *p ≤ 0.05, **p ≤ 0.01, NS: no significance.

Studies show that DLL4 is selectively expressed by intratumoral endothelial cells in breast cancer but not in normal breast tissue^31^, and aberrant activation of Notch signaling is associated with breast carcinogenesis^32^,^33^. To evaluate the *in vivo* anti-tumor activity of H_3_L_2_, a MDA-MB-231 breast cancer xenograft model was used. After implantation, tumor cells were allowed to grow for about 10 days before the tumor volume reached 100 mm^3^. Treatment of H_3_L_2_ on these tumor-bearing mice obtained maximal tumor inhibition rates of 35% and 59% at dose of 2.5 mg/kg and 5 mg/kg respectively. As a reference for humanized antibody, 5 mg/kg chimeric antibody H_C_L_C_ was shown to achieve the similar tumor inhibition rate (56%) with the same dose of H_3_L_2_ (*P* = 0.406) (Fig. 5a,b).

The effect of H_3_L_2_ on mitotic index (Ki67) and apoptosis (cleaved caspase-3) in the tumor was detected by immunohistochemical staining. A distinct increase in cleaved caspase-3 and a reduction of Ki67 level were observed with treatment of H_3_L_2_, which indicates that H_3_L_2_ inhibited tumor cells proliferation and induced apoptosis of tumor cells (Fig. 5e,g). Angiogenesis in the tumor was further analyzed by immunofluorescence, where treatment of H_3_L_2_ result in increase in vessel number, along with a reduction in percentage of α-SMA-positive (α-SMA+) mural cells (Fig. 5f,h). This result was consistent with H_3_L_2_ caused endothelium cell proliferation, while lack of mural cells led to immature vessels^34^,^35^ (CD31+/α-SMA−). Different from H_3_L_2_ induced increment of neoangiogenic vessels, tumors treated with vehicle control showed less vessels but all coated with mural cells (CD31+/α-SMA+). Reduction of NICD levels in the same tumor samples were observed in Western Blot assay (Fig. 5c,d). In conclusion, H_3_L_2_ demonstrated an anti-tumor effect *in vivo* through promotion of dysfunctional angiogenesis and cell apoptosis in tumor.

## Discussion

The aim of this study is to humanize a murine antibody MMGZ01 targeting DLL4 and evaluate its anti-tumor activity. The ultimate purpose of humanization is to substitute murine residues with human residues to reduce immunogenicity while retaining the original binding affinity to the maximum. The antigen binding affinity mainly depends on six CDRs which are shaped and supported by the relatively conserved FRs in the variable regions. CDR grafting from murine to human FR templates with highest similarity is a widely used method for humanization. In this study, the optimal human FR templates were screened by structure-based computational method. Studies show that chimeric antibody usually shares the similar binding affinity with the original murine antibody^16^,^36^. Meanwhile, the human constant regions of chimeric antibody make it more convenient for detection in parallel experiments, so we designed and generated a chimeric antibody (H_C_L_C_) as positive reference for humanized antibodies.

Firstly, a precise MMGZ01 Fv structure was obtained based on antibody structure templates for all subdomains (CDR1-3 and FR1-4). The sequence similarities and structure fitness of templates were comprehensively scored by MOE (Fig. 1a, Table 1). According to the Fv structure, canonical residues were identified and categorized into 3 types: VH-VL interface core residues which were important for dimer packing; CDR loop foundation residues buried directly underneath the CDRs^23^ and CDR loop interaction residues directly contacted with the CDR residues (hydrogen, hydrophobic or ion bond) (Table 2). These crucial residues were considered to be retained from the murine antibody in CDR grafting. The antigen epitope recognized by MMGZ01 was screened from a dodecapeptide phage display library, and selected in DLL4 before docking with MMGZ01 Fv. The complex structure predicted through docking approach provided important residues interactions in the antibody-antigen binding site. Further the interactive residues in MMGZ01 Fv were also recommended to be retained in humanization. Moreover, the interactive residues in CDRs may be designated as specificity-determining residues (SDRs), and used for SDRs grafting. This new approach of antibody humanization may further reduce the anti-variable region or anti-idiotypic responses caused by xenogeneic CDR^37^, and is our plan for future work.

The humanization of MMGZ01 was started with CDR grafting. Human V or J genes with highest sequence similarity and most canonical residues conserved were chosen for CDR grafting. Although most canonical residues were conserved, there were still some differed (Fig. 2, Table 2), and evidence showed that the CDR grafting antibody was not able to retain the antigen binding activity (Supplementary Fig. S3). To evaluate the contribution of these differed canonical residues to antigen binding, we introduced a number of back mutations to the CDR grafting antibody (see Supplementary Material). The humanized antibody (H_3_L_2_) which back mutated all the 12 residues showed similar antigen binding capacities with the chimeric antibody H_C_L_C_ (Supplementary Fig. S3). Meanwhile, 3D structure of H_3_L_2_ was also built and superposed with the MMGZ01 Fv structure. The calculated structural RMSD (0.824 Å) indicated a similar conformation between the two antibodies. Experimental results were consistent with the computational predictions, thus we confirmed the importance of FRs canonical residues identified here. In addition, the efficient and reliable approach for humanization proposed in this study, which combined computational modeling with experiments, could be applied to the humanization of other antibodies.

Stable transfectants expressing H_3_L_2_ were introduced by electroporation and screened by G418. The purified H_3_L_2_ showed expected molecular weight in reduced and non-reduced condition, indicating an integrated assembly of the antibody (Fig. 3a,b). To investigate the binding specificity and affinity of H_3_L_2_ to DLL4, we used the SPR-based method which provide sensorgrams visualizing all the data from an interaction in real time. Our results indicates that H_3_L_2_ retained the high specificity and affinity from its parental antibody (Fig. 3c,d).

Moreover, the effects of H_3_L_2_ on DLL4 positive cells were investigated with HUVEC cells. As a normal endothelium cell line, HUVEC expresses relatively lower level of DLL4 compared to tumor vessels^28^. We found that addition of rhDLL4 inhibited proliferation of HUVEC cells, while H_3_L_2_ effectively reversed this inhibition (Fig. 4b). Furthermore, NICD, as the activated form of Notch receptor, was significantly decreased in the treatment of H_3_L_2_ (Fig. 4c,d). In conclusion, the humanized antibody H_3_L_2_ effectively blocked the DLL4-Notch signaling pathway and further promoted the proliferation of HUVEC cells *in vitro*.

DLL4 is unregulated by intratumoral endothelial cells in most breast cancer^31^, and aberrant activation of Notch signaling is associate with breast carcinogenesis^32^,^33^. When endothelial cells form the inner vessel wall, mural cells associate with and coat the endothelial cell tube to make mature and functional vessels^34^,^35^. In this study, a MDA-MB-231 xenograft model was established to evaluate the *in vivo* anti-tumor activity of H_3_L_2_. The results indicates that H_3_L_2_ inhibited tumor growth through blockage of DLL4-Notch signaling pathway (Fig. 5a–d). In the presence of H_3_L_2_, increase in vessel number along with a reduction in percentage of mural cells was observed (Fig. 5f,h). Thus, the vessels induced by H_3_L_2_ were immature and dysfunctional, which contributed to the inhibition of tumor growth. In addition, the observed increase in expression of cleaved caspase-3 along with decrease in Ki67 (Fig. 5e,g) indicates that H_3_L_2_ mediated tumor inhibition was associated with induction of tumor cell apoptosis and inhibition of tumor cell proliferation.

In summary, H_3_L_2_ is an ideal humanized antibody that blocks DLL4/Notch pathway against breast carcinoma *in vivo* and it is a promising anti-tumor drug candidate for clinical studies.

## Methods

### Materials

Restriction enzymes were purchased from NEB (New England biolabs, Ipswich, MA, USA). T4 DNA Ligase was purchased from Thermo Scientific (Shanghai, China). Eukaryotic expression vectors pMH3-H, pCApuro-H, pMH3-L, pCApuro-L contained full-length IgG1 H & L chain were preserved in our lab^27^. Chinese hamster ovary (CHO-s) cell line was purchased from AmProtein (Hangzhou, Zhejiang, China) and cultured in DMEM/F12 medium with 10% (v/v) fetal bovine serum (FBS). HUVEC cells purchased from ScienCell research laboratories (Carlsbad, CA, USA) were c\ultured in ECM medium supplement with 5% (v/v) FBS and 1% (v/v) ECGS (ScienCell, San Diego, CA, USA). Cell culture media and trypsin powder were purchased from Life Technologies (Basel, Switzerland).

### Cloning of VH and VL genes of MMGZ01

The total RNA of MMGZ01 hybridoma cell line was isolated using a Trizol reagent (Invitrogen, Burlington, ON, Canada), and reverse-transcribed to cDNA with a M-MuLV RT-PCR kit (Sangon Biotech, Shanghai, China). In the Polymerase Chain Reaction (PCR), PrimeSTAR HS DNA polymerase was used to amplify the VH and VL genes with designed degenerate primers specific for murine antibodies (Takara Bio, Dalian, Liaoning, China). Chains of VH and VL were detected in a 1% agarose gel and corresponding bands were purified using an Agarose Gel Extraction Kit (Takara Bio, Dalian, Liaoning, China). The purified chains were then cloned to pMD18T vector for sequencing (Genscript Corporation, Nanjing, Jiangsu, China).

### Antibody modeling and evaluation

The 3D structure model of MMGZ01 Fv was built by antibody homology modeling method^38^ using chemical computing and molecular modeling software, Molecular Operating Environment (MOE), version 2013.08, where we searched antibody structure templates in PDB database for each subdomains. Templates for FRs in VH & VL or the VH-VL dimer were searched by sequence identity, and the CDR regions by sequence similarity.

The Fv model of MMGZ01 was built with the Antibody Builder according to the templates found, then refined to relive strained geometry and further applied energy minimization scheme in Amber 10:EHT force field and achieved a final structure model^39^. This resulting Fv structure was tested on stereo-chemical quality with Protein Geometry in MOE. Backbone Bond lengths, angles and dihedrals were compared to averages and standard deviations of a reference database of high resolution structures from the PDB^18^.

As the precise structure of Fv was established, we further identified the canonical residues in FRs which were important for CDR conformation maintaining or the binding affinity of antibody. These canonical residues could be classified into 3 types: the VH-VL interface core residues^23^ (distance = 5.00 Å, buried percentage >85%), the CDR loop foundation residues (radus = 5.00 Å, buried percentage >85%) and the CDR loop interaction residues (cutoffs = 5.00 Å).

### Epitope mapping by randomized dodecapeptide phage display library

Epitope mapping of MMGZ01 antibody was directed with the Ph.D.™-12 Phage Display Peptide Library Kit (New England Biolabs, Ipswich, MA, USA). In the panning, 1.5 × 10^10^ phages were incubated with a plate coated with MMGZ01 for 2 h at 37 °C, after washing away the unbound phages, the specifically bound phages were eluted with Glycine - HCl (pH 2.2) and amplified in *E. coli* ER2738 in the logarithmic phase for another binding - amplification cycle. After 4 cycles, individual clones were amplified and characterized by ELISA. Detected with HRP-conjugated anti-M13 monoclonal antibody (Sino Biological Inc. Beijing, China), the DNA of positive clones was isolated and sequenced by Genscript Corporation (Nanjing, Jiangsu, China).

### Molecular docking

The structure of DLL4 were downloaded from SWISSMODEL. Epitope recognized by MMGZ01 were selected as contact residues in DLL4 before docking. A rigid protein docking program ZDOCK^21^,^22^ sever (version ZD3.0.2) was used, and MMGZ01 Fv was set as receptor structure, DLL4 was set as ligand structure. The top modeled complex structure was analyzed by MOE.

### Humanization of MMGZ01 Fv

The humanization of MMGZ01 Fv contained two steps of design: First, grafted the CDR regions onto the FR regions of human immunoglobulin germline V and J gene to get a CDR grafted Fv; Second, chose the changed canonical residues in FR regions to back mutate to the original murine ones. We searched the human immunoglobulin germline V genes for templates of FR1-3. The human donor V genes with highest FR sequence similarity and most canonical residues conserved were selected. Human donor J genes provided for templates of FR 4 were selected based on their highest VH & VL CDR3 similarity and canonical residues in FR4. Then, a CDR grafted Fv (VH_g_, VL_g_) was archived by grafting MMGZ 01 VH & VL CDRs onto FRs of human donors V and J genes.

Although the most canonical residues conserved templates were selected, there were some canonical residues changed, which were candidates for back mutation. The humanized Fv based on CDR grafting and back mutation with all the candidates was named as H_3_L_2_ Fv (VH_3_, VL_2_).

### Construction and transient expression of chimeric or humanized antibodies

The VH & VL of MMGZ01 or humanized antibodies were added Kozak sequence, signal peptide and restriction enzyme cutting sites, then optimized to CHO-preferred codons, synthesized and cloned to pUC57-simple vectors by Genscript Corporation (Nanjing, Jiangsu, China) respectively. Restriction enzyme cutting site in VH were *EcoR*I & *Nhe*I, and in VL were *EcoR*I and *Sal*I. Expression vectors (pMH3-H, pCApuro-H, pMH3-L, pCApuro-L) with the corresponding cutting site between VH and CH or between VL and CL^27^ were used. After double digestion and ligation of these vectors, VH & VL of MMGZ01 or humanized antibodies were cloned to pMH3 and pCA-puro replacing VH & VL of IgG1 to join full-length chimeric (H_C_L_C_) or humanized antibodies. Nucleotide sequences of recombinant H-chain and L-chain vectors were confirmed with sequencing by Genscript Corporation (Nanjing, Jiangsu, China).

The recombinant expression vectors of H_C_L_C_ or humanized antibodies were then transient transfected into CHO-s cells using SuperFectin II Transfection Reagent (Pufei Biotech Co., Ltd, Shanghai, China). After 72 h’ incubation, the supernatant was collected and analyzed by ELISA (see Supplementary Material).

### Structure superposition and RMSD calculation

The 3-D structure model of H_3_L_2_ Fv was built and superposed with the structure model of MMGZ01 Fv. Structure RMSD between corresponding protein alpha carbons (Cα) were calculated in MOE, where a weighted non-linear optimization was used to determine the solid-body transformations required to maximize the superposition of the protein atomic coordinates^26^.

### Stable expression and purification of H_C_L_C_ or H_3_L_2_

Expression vectors of H_C_L_C_ or H_3_L_2_ were transfected to CHO-s cells by electroporation and stable transfectants were screened using the method previously described^27^. Clones producing highest amount of antibodies were grown in medium with 5% FBS and purified by Protein A affinity chromatography (GE Healthcare, Buckinghamshire, UK).

### Affinity analysis

The binding kinetics of H_C_L_C_ or H_3_L_2_ to rhDLL4 were analyzed using an Surface Plasmon Resonance (SPR)-based assay on Biacore X100 system (GE Healthcare, Buckinghamshire, UK). An anti-human IgG Fc antibody (GE Healthcare, Buckinghamshire, UK) was firstly immobilized onto a CM5 biosensor chip. Then, appropriate concentration of H_C_L_C_ or H_3_L_2_ was captured to the surface with an increase of Resonance Unit (RU) up to 2,000. Finally, various concentrations of rhDLL4 were passed through the chip. After each reaction, the captured H_C_L_C_ or H_3_L_2_ antibody and analyte were removed by Regeneration (3 M magnesium chloride). The whole reaction was conducted at 25 °C and flow rate of 30 μl/min. Sensorgrams of each concentration were obtained and analyzed by Biacore evaluation software (GE Healthcare, Buckinghamshire, UK). The equilibrium constant *K*_*D*_ was calculated from ratio of dissociation rate constant *k*_*d*_ to association rate constant *k*_*a*_ (*k*_*d*_/*k*_*a*_).

### Flow cytometry

4 × 10^5^ HUVEC cells cultured in ECM were digested by trypsin and resuspended in PBS containing 2% FBS. Then the cells were washed and incubated with 10 μg/ml HcLc or H_3_L_2_ at 4 °C for 1 h, followed by FITC conjugated donkey-anti-human IgG H+L (Sangon Biotech, Shanghai, China). Cells incubated with secondary antibody were used as background fluorescence control. 1 × 10^4^ cells were analyzed in all individual detections. The binding assay was performed on a BD FACS flow cytometry and the obtained data was processed using FlowJo 7.6 software.

### Cell proliferation assay

96-well plates immobilized with 1μg/ml rhDLL4 or PBS were seeded with 4 × 10^3^ HUVEC cells, meanwhile, series concentrations of H_C_L_C_ or H_3_L_2_ were added into the plates, each concentration has three repeats. Same volume PBS was added as control. After incubation for 72 h, cell viability was determined by MTT assay.

### Notch signaling blockage analyzed by Western Blot

6-well plate immobilized with 1 μg/ml rhDLL4 was seeded with 5 × 10^5^ HUVEC cells, meanwhile, H_C_L_C_ or H_3_L_2_ were added to final concentrations of 0.1, 1,10 μg/ml. After incubation for 24 h, the cells were lysed and extracts were harvested by RAPI buffer (Beyotime, Shanghai, China). About 20 μg of extracts were resolved by electrophoresis and then transferred onto PVDF membranes. The membranes were then blocked and incubated with cleaved Notch1(Val1744) antibody (Cell Signaling Technology, Boston, MA, USA) or anti-β-actin (Anbo Manufacturing, Colville, WA, USA), followed by incubation of HRP conjugated goat-anti-rabbit antibody (Signalway Antibody LLC, College Park, MD, USA). Finally, after reacting with ECL substrates, the membrane was analyzed by Bio-Rad GelDoc ^TM^ XR^+^ system.

### Human tumor xenograft model and drug administration

The human breast cancer cell line MDA-MB-231 was obtained from ATCC and the cells were cultured in DMEM medium supplement with 10% FBS. 1 × 10^7^ MDA-MB-231cells suspended in 100 μl PBS were subcutaneously injected into the left flank of 8-week old male nude mice. When the tumor volume reached about 100 mm^3^, the tumor-bearing mice were randomized and divided into groups for control (PBS) and different doses of H_3_L_2_ (low dose: 1 mg/kg, middle dose: 2.5 mg/kg and high dose: 5 mg/kg), each group contained 6 mice. 5.0 mg/kg chimeric antibody was used as comparison for H_3_L_2_ and antibodies were intravenously administrated every other day. Meanwhile, tumor volumes were measured every third day using a vernier caliper applying the formula V = LW^2^/2 (L is the long axis and W is the short axis of the tumor).

On the final day of study, percent (%) inhibition rates (IR) were calculated as IR% = 100 × (1 − Vt/Vc), where Vt is average elevated volume of drug-treated group, and Vc is average elevated volume of control group. After 3 weeks’ treatment, the mice were sacrificed and the tumors were prepared for analysis. All experiments were approved by the Animal Ethics Committee and were performed in accordance with the animal experiment center guidelines of Comparative Medicine Center of Yangzhou University.

### Immunohistochemical staining and immunofluorescence

4% paraformaldehyde fixed, paraffin embedded tumor tissues were cut to 5 μm thick sections for immunohistochemical (IHC) staining or immunofluorescence (IF). After rehydration, antigen retrieval and endogenous peroxidases quenching, the sections were blocked and further incubated with primary antibodies followed by corresponding secondary antibodies conjugated with HRP (for IHC) according to the instruction of Vectastain ABC Kit (Dako, Copenhagen, Denmark). The primary antibodies used were anti-Ki67, anti-cleaved caspase-3, anti-CD31 and anti-α-SMA (Cell Signaling Technology, Boston, MA, USA). For IF, cell chromosome was stained with 4′,6-diamidino-2-phenylindole (DAPI) and shown as blue. In addition, CD31-positive cells were shown as red, and α-SMA-positive were shown as green. Tumor sections were viewed and photographed using the Zeiss Axio Vert A1 microscope (Carl Zeiss, Thornwood, NY, USA).

### Statistical analysis

Statistical analysis and variable slopes were performed with GraphPad prism software (GraphPad software Inc., La Jolla, CA, USA). Significance levels were estimated using the two-tailed student’s t test and *P* values of 0.05 or less were considered statistically significant.

## Additional Information

**How to cite this article**: Jia, X. *et al*. A humanized anti-DLL4 antibody promotes dysfunctional angiogenesis and inhibits breast tumor growth. *Sci. Rep.* **6**, 27985; doi: 10.1038/srep27985 (2016).

## Supplementary Material

Supplementary Information

## Figures and Tables

**Table 1:** Structure templates for MMGZ01 Fv homology modeling.

Regions	Templates (PDB ID)	Identity or Similarity (%)	Structure score
LFRs	1E6O. L	87.0	94.6
L-CDR1	3LS5. L	89.7	91.9
L-CDR2	2BRR. X	88.8	95.8
L-CDR3	4ETQ. B	83.7	94.4
HFRs	1E6O. H	89.2	94.7
H-CDR1	1IQW. H	82.3	79.1
H-CDR2	3CMO. Y	83.2	70.2
H-CDR3	3UPC. F	45.6	56.4

The overall backbone integrity of each antibody subdomain was assessed by the Structure score, below 50 of which indicates possibilities of structural issues.

**Table 2:** Canonical residues in FRs of VH & VL for back mutation.

Position	Residues	Interface core	Loop foundation	Loop interaction
LFR1	I4	0	1	1
LFR2	P47	1	1	0
LFR2	W48	0	1	0
LFR2	Y50	1	1	1
LFR3	Y72	0	1	1
HFR1	Y27	0	1	0
HFR2	I37	1	1	1
HFR2	K38	1	1	0
HFR2	I48	0	1	1
HFR3	A68	0	1	0
HFR3	L70	0	1	1
HFR3	G98	0	1	0

**1** represents yes and **0** represents no, residues shown are original murine ones.
