# Supplementary material for: A humanized anti-DLL4 antibody promotes dysfunctional angiogenesis and inhibits breast tumor growth
Source: Sci Rep. 2016 Jun 15;6:27985. doi: 10.1038/srep27985 (PMC4908374; doi:10.1038/srep27985)
Supplement: Supplementary Information [file srep27985-s1.pdf]

# A humanized anti-DLL4 antibody promotes dysfunctional angiogenesis and inhibit breast tumor growth

**Xuelian Jia[1], Wenyi Wang[1, 2], Zhuobin Xu[1], Shijing Wang[1], Tong Wang[1], Min Wang[1, *], Min Wu[1, *]**

[1] State Key Laboratory of Natural Medicines, School of Life Science & Technology, China Pharmaceutical University, Nanjing, Jiangsu 210009, China

[2] The Rutgers Center for Computational and Integrative Biology, Rutgers University, Camden, NJ 08102, United States

The authors Xuelian Jia and Wenyi Wang contributed equally to this work.

[*] Corresponding authors. Address: China Pharmaceutical University, No. 24 Tong Jia Xiang, Nanjing, Jiangsu 210009, China.

Tel.: +86 25 8327 1395. Fax: +86 25 8327 1395.

Email Address: minwang@cpu.edu.cn (Min Wang), mickeywu2001@163.com (Min Wu).

**Introduction of back mutations to the CDR grafting**

Canonical residues belonged to all the 3 types (e.g. VL-Y50, VH-I37) and/or with dStability most positive (e.g. VL-Y50, VL-Y72, VH-Y27, VH-I48, VH-I37) (see **Table. S1**) were back mutated with priority and the rest residues were gradually added in the later version. We designed 3 versions of back mutate VH (named as $VH_1$,$VH_2$ and $VH_3$) and 2 versions of back mutant VL ($VL_1$, $VL_2$), the amino acid sequences of which were shown in Fig. S2. $VH_1$ mutate 27G, 37V, 48M to the original murine residues, $VH_2$ further mutate 38R and 70I while VH3 mutate all the 7 residues. Similarly, $VL_1$ mutate 50K, 72F and $VH_2$ mutate all the 5 residues to the original murine one. The 3 mutate VH and 2 mutate VL were combined to make 6 humanized antibodies ($H_1L_2$, $H_2L_1$, $H_3L_1$, $H_1L_2$, $H_2L_2$ and $H_3L_2$).

**Enzyme-linked immunosorbent assay (ELISA)**

ELISA was performed to test antigen binding activity of $H_CL_C$ or humanized antibodies, 1 μg/ml rhDLL4 was immobilized to 96-well plates at 4 ℃ overnight, and then incubated with a series concentrations of purified culture supernatant at 37 ℃ for 1 h. Supernatant of non-transfected cells was used as vehicle control. Plates were washed and incubated with HRP conjugated goat-anti-human IgG H+L as detecting antibody. Lastly, antibody bound to the plate was determined by monitoring the absorbance difference between 450 nm and 630 nm in BioTek Synergy 2 plate reader.

These 6 humanized antibodies were constructed and expressed in CHO-s cells. As shown in Fig. S3, the affinity of humanized antibodies were analyzed by ELISA. The binding affinity of the CDR grafted antibody HgLg decreased, but was recovered by back mutation ($H_3L_2$). While the rest antibodies failed to recover the binding affinity and $H_2L_1$ or $H_2L_2$ even lost the binding activity.

| | Murine (wild type) | Mutant | Canonical type | dStability(Kcal/mol) |
|---|---|---|---|---|
| **VL** | I4 | L4 | 2, 3 | -0.0128 |
| | P47 | L47 | 1, 2 | -2.1287 |
| | W48 | L48 | 2 | 0.6853 |
| | Y50 | K50 | 1, 2, 3 | 3.1368 |
| | Y72 | F72 | 2, 3 | 1.2154 |
| **VH** | | | | |
| | Y27 | G27 | 2 | 4.1383 |
| | I37 | V37 | 1, 2, 3 | 0.8707 |
| | K38 | R38 | 1, 2 | 0.7352 |
| | I48 | M48 | 2, 3 | 1.0759 |
| | A68 | V68 | 2 | -1.6471 |
| | L70 | I70 | 2, 3 | -0.9504 |
| | G98 | R98 | 2 | -1.2529 |

**Table S1. Antibody structure stability change upon mutation.** Canonical type: 1 represents VH-VL interface core residues; 2 represents CDR loop foundation residues; 3 represents CDR loop interaction residues.

| Regions | Templates (PDB ID) | Identity or Similarity (%) | Structure score |
|---|---|---|---|
| **LFR** | 4KQ3. L | 66.3 | 95.9 |
| **L-CDR1** | 3LS5. L | 89.7 | 91.9 |
| **L-CDR2** | 1WC7. A | 88.8 | 78.3 |
| **L-CDR3** | 4ETQ. B | 83.7 | 94.4 |
| **HFR** | 4KQ3. H | 90.5 | 95.9 |
| **H-CDR1** | 4OTX. I | 89.3 | 97.0 |
| **H-CDR2** | 3ET9. FH | 81.2 | 72.9 |
| **H-CDR3** | 3UPC. F | 45.6 | 56.4 |

**Table S2. Structure templates for $H_3L_2$ Fv homology modeling.** The overall backbone integrity of each antibody subdomain was assessed by the Structure score, below 50 of which indicates possibilities of structural issues.

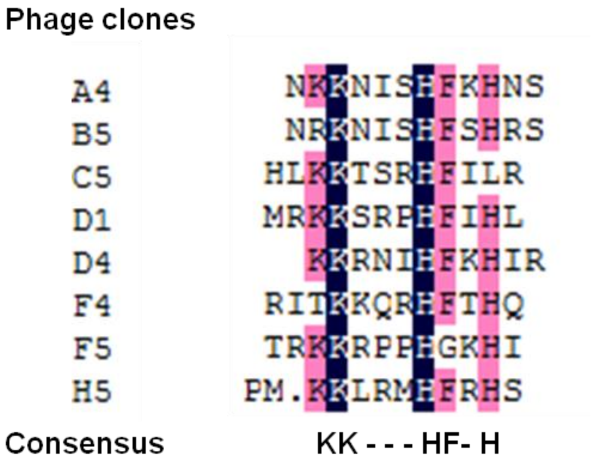


**Fig S1. Epitope mapping of MMGZ01.** A dodecapeptide phage display library was screened against MMGZ01. The consensus residues between the positive clones were KK---HF-H. Residues with 100 % identity are marked black, and residues with or over 75 % identity are marked purple.

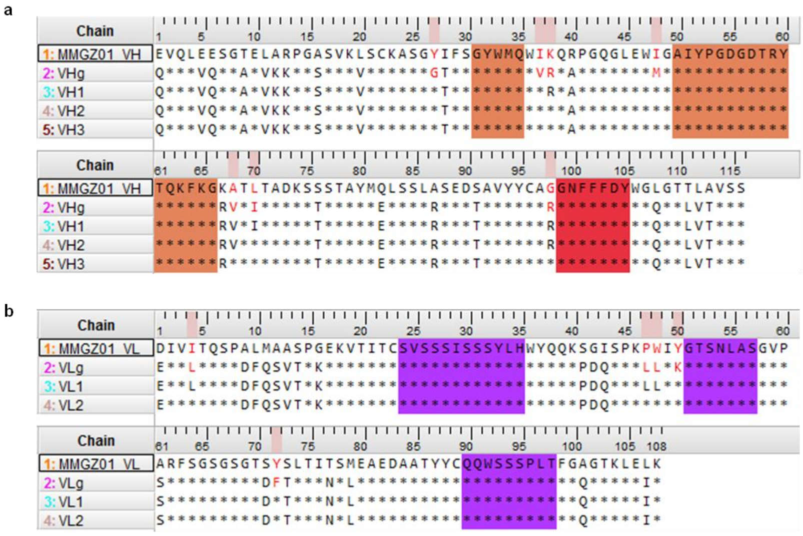


**Fig S2. Amino acid sequences alignment of back mutate antibodies.** (**a**) VH and (**b**) VL. Canonical residues differed between MMGZ01 and the CDR grafted antibody were marked as red. * represents residues that were identical to the corresponding residues of MMGZ01.

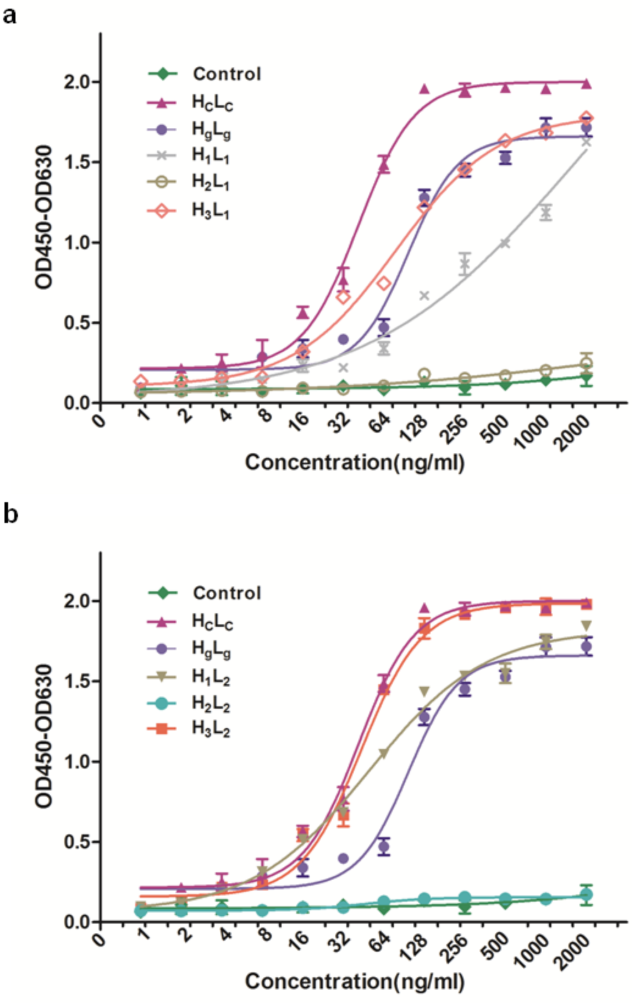

**Fig S3. Antigen binding capacity of back mutate antibodies analyzed by ELISA.** The chimeric antibody named as $H_CL_C$ was used as reference of binding affinity. (**a**) Binding curves of $H_1L_1$, $H_2L_1$ and $H_3L_1$. (**b**) Binding curves of $H_1L_2$, $H_2L_2$ and $H_3L_2$. Data are shown as mean ± SD, n = 3.
